# Characteristics of the Stool, Blood and Skin Microbiome in Rosacea Patients

**DOI:** 10.3390/microorganisms12122667

**Published:** 2024-12-23

**Authors:** Marie Isolde Joura, Antal Jobbágy, Zsuzsanna A. Dunai, Nóra Makra, András Bánvölgyi, Norbert Kiss, Miklós Sárdy, Sarolta Eszter Sándor, Péter Holló, Eszter Ostorházi

**Affiliations:** 1 Department of Dermatology, Venerology and Dermatooncology, Semmelweis University, 1085 Budapest, Hungary; 2 Károly Rácz Doctoral School of Clinical Medicine, Semmelweis University, 1085 Budapest, Hungary; 3 Institute of Medical Microbiology, Semmelweis University, 1089 Budapest, Hungary; 4 Department of Dermatology, Pál Heim National Institute of Pediatrics, 1089 Budapest, Hungary

**Keywords:** rosacea, stool microbiome, blood microbiome, skin microbiome, 16S rRNA, metabolic pathways

## Abstract

Several research groups have confirmed that in the pathogenesis of the chronic inflammatory skin disorder rosacea, the composition of the skin and fecal microbiome of affected patients differs from that of healthy individuals. We studied the stool, blood and skin microbiomes of rosacea and control patients using 16S rRNA sequencing. Our goals were to determine 1. whether the microbiome characteristics of rosacea patients differ from that of healthy individuals, 2. whether the change experienced on the skin can be confirmed by alterations in the stool microbiome through the mediation of the blood and 3. whether the metabolic activity of the changed skin, blood or fecal microbiome can play a role in the pathogenesis of rosacea. The rosacea skin microbiome differed significantly from the healthy skin microbiome in both alpha and beta diversity, as well as in the abundance of the genera. Only a few genera abundances differed significantly in stool and blood samples. The most significant representatives of the rosacea skin microbiome, *Staphylococcus*, *Cutibacterium*, *Corynebacterium* and *Neisseria*, cannot be derived from the feces or blood. The metabolic pathways associated with healthy fecal microbiome contributed to the production of anti-inflammatory short-chain fatty acids. While the increased production of adenosylcobalamin, L-isoleucine and thiazole by the microbiome of healthy skin appeared to have a protective effect, the excessive heme and H_2_S production experienced in rosacea skin likely contribute to the deterioration of the pathology.

## 1. Introduction

Rosacea is a chronic inflammatory condition primarily affecting the facial region, characterized by symptoms such as redness, flushing and sometimes pustules. Depending on the phenotype, it is characterized by erythema, telangiectasia, papules and pustules or hyperplasia of the connective tissue and sebaceous glands. Middle-aged women with light skin type are most frequently affected [1]. Rosacea is classified into four clinical subtypes: (1) erythematotelangietic rosacea (ETR), (2) papulopustular rosacea (PPR), (3) phymatous rosacea and (4) ocular rosacea [2]. The usual classification based solely on morphological characterization is not really optimal. Rosacea presents with overlapping clinical subtypes [3]. The exact pathogenesis of the disease is unknown, but genetic predisposition, environmental factors, neurovascular reactivity and an overactive immune response are considered possible triggers [4]. Various factors such as sun exposure, stress, hot drinks and spicy food can exacerbate the symptoms [5,6]. An association between rosacea and gastrointestinal diseases, including irritable bowel syndrome, inflammatory bowel disease, small intestine bacterial overgrowth or *Helicobacter pylori* infection is demonstrated in population-based studies [7,8]. Topical antibiotics that only affect the skin microbiome, systemic antibiotic treatment or probiotics that affect the composition of the gut microbiome, can also be effective in curing rosacea [9,10,11]. While the exact etiology of rosacea remains unclear, recent research has highlighted the significant role of the microbiome—both gut and skin—in its pathogenesis [12,13,14,15,16]. Having said that, these results have been inconsistent and remain inconclusive. To our best knowledge, only one publication investigated the composition of the blood microbiome of rosacea patients [17]. Changes in the fecal microbiome can have a direct effect on the pathogenesis of rosacea, if the characteristic taxon abundance changes in the stool also occur in the microbiome composition of other parts of the body. For example, research in psoriasis patients found that they actually had increased levels of bacterial DNA in the bloodstream compared to healthy controls, and it was presumed that the bacterial DNA would have originated from the intestinal lumen [18]. Also, an indirect effect can result from products of the metabolic activity of the altered fecal microbiome entering the blood [19]. Dysbiosis characterized by high levels of H_2_S-producing bacteria can lead to increased gut permeability (“leaky gut”), allowing pro-inflammatory substances to enter systemic circulation and affect skin conditions [20]. The development of molecular genetic tests in recent years made it possible not only to examine the composition of the microbiome of a specific anatomical area but also to infer biochemical activities from the microbial composition [21].

With our current research, we intended to clarify whether (1). there is a difference in the microbiome composition of the skin, blood and feces of our rosacea patients and healthy controls, (2). whether the change experienced on the skin can be confirmed by the change in the stool microbiome through the mediation of blood and (3). Whether the metabolic activity of the changed skin, blood or fecal microbiome can play a role in the pathogenesis of rosacea.

## 2. Materials and Methods

### 2.1. Ethical Considerations

Sample collection protocols were approved by the Ethics Committee of Semmelweis University (SE RKEB: 282/2020). The study was conducted in accordance with the Declaration of Helsinki’s ethical standards that promote and ensure respect and integrity for all human subjects. All study participants provided written informed consent and that data from their personal test results could be published. Data and test results in the manuscript cannot be linked to individual participants because all tests were anonymized.

### 2.2. Sample Collection

Our recent microbiome molecular investigation compared the stool, blood and skin samples of 18 rosacea patients in addition to stool, blood and skin samples of 9 healthy individuals. From February to August 2021, the study included untreated patients who were first diagnosed with rosacea at the Outpatients Clinic of Department of Dermatology, Venerology and Dermatooncology of Semmelweis University during this period. The inclusion criterion was the first diagnosis of rosacea. Exclusion criteria were pregnancy, previous use of probiotics or antibiotics in the last 6 months, gastrointestinal disease or complaints in the last 4 weeks and any topical or systemic treatment of a skin disorder in the last 4 weeks. We selected nine healthy volunteers of similar age and gender distribution at the same time as the control group. The characteristics of the study participants are presented in Table 1.

At least 3-3 mL of whole blood was collected into citrate filled VACUETTE collection tubes (Greiner Bio-One, Stonehouse, UK), fecal and skin swab samples were col-lected in Zymo DNA/RNA Shield (Zymo Research Corp., Irvine, CA, USA). Prior to skin sample collection from the bilateral cheeks, no washing was permitted for 24 h. All the samples were stored at −80 °C until DNA extraction. 

### 2.3. DNA Isolation, 16S rRNA Gene Libary Preparation and MiSeq Sequencing

From skin and stool samples, DNA isolation was performed using a ZymoBIOMICS DNA miniprep kit (Zymo Research Corp., Irvine, CA, USA). DNA isolation from blood samples was performed using a NucleoSpin blood, mini kit (Macherey-Nagel, Allentown, PA, USA) according to the instructions of the manufacturer. The V3-V4 region of bacterial 16S rRNA gene was amplified with tagged primers. PCR and DNA purifications were performed according to Illumina’s protocol. PCR product libraries were assessed using a DNA 1000 kit with an Agilent 2100 bioanalyzer (Agilent Technologies, Waldbronn, Germany). Illumina MiSeq platform (Illumina, San Diego, CA, USA) and MiSeq Rea-gent Kit v3 (600 cycles PE) were used to sequence the equimolar concentrations of pooled libraries.

Extraction negative controls and PCR negative controls were included in every run. All analysis procedures were conducted in duplicate from 2 separately isolated DNA samples from each patient. Raw sequencing data were retrieved from Illumina BaseSpace, and data were analyzed using the CosmosID [22] bioinformatics platform. The Cos-mosID-HUB Microbiome’s 16S workflow for taxonomy and species-level identification was conducted with DADA2’s naive Bayesian classifier, using the Silva version 138 database.

### 2.4. Statistical Analysis

The levels of statistical significance (*p* < 0.05) for the difference between bacterial taxa abundances measured in the different cohorts were calculated via the Mann–Whitney U test. Statistical significance between cohorts was implemented via Wilcoxon rank sum test for Chao1 alpha diversity and PERMANOVA analysis for Bray–Curtis principal coordinate analysis (PCoA) beta diversity using the statistical analysis support application of CosmosID [22]. In addition, the CosmosId bioinformatics platform was used for LEfSe (linear discriminant analysis effect size) to identify features including taxa and metabolic pathways, characterizing the differences between the two cohorts (Cosmosid Inc., Germantown, MD, USA).

## 3. Results

We found few bacterial 16S rRNA read numbers in four blood samples of rosacea patients and in one control blood sample. At the same time, human-derived mitochondrial DNA was amplified in these samples. All samples from these individuals were excluded from the comparative studies. A sufficient amount of read numbers in stool, blood and skin samples from the fourteen rosacea and eight control patients were collected to compare and evaluate the data. The median number of reads within one sample, regardless of the sample type was 186,792 (IQR: 16,548).

**Figure 1:**
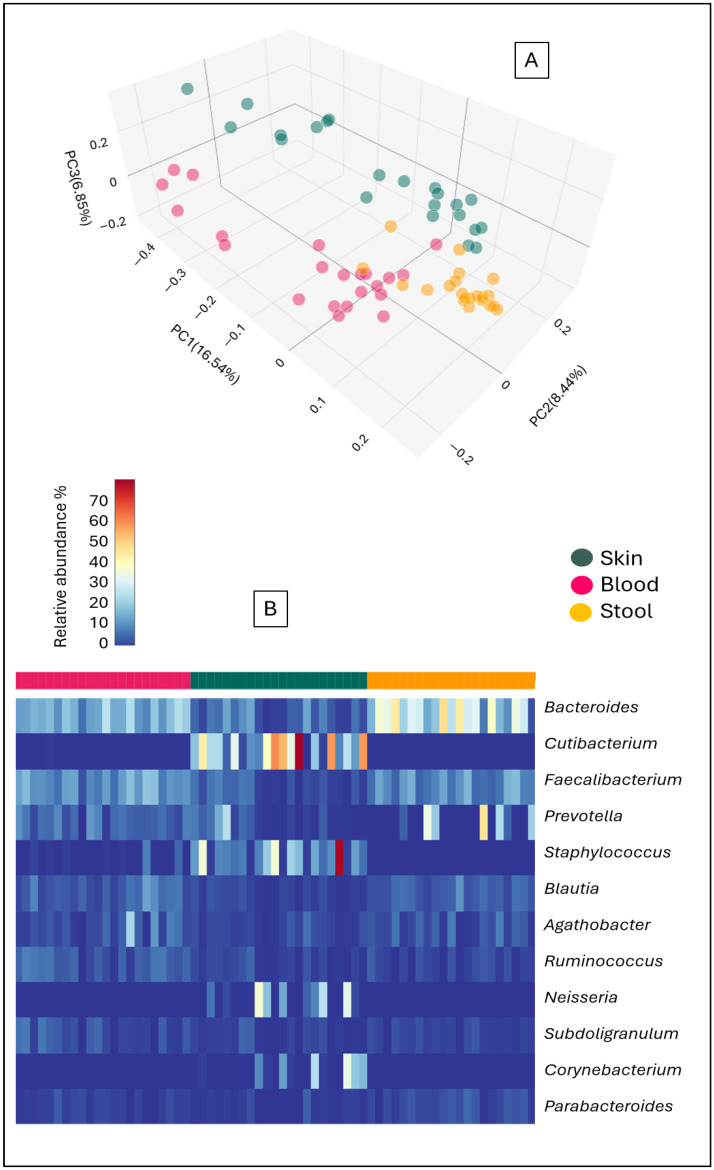
Principal component analysis (**A**) and heatmap (**B**) of bacterial abundance of stool, blood and skin samples of the rosacea patients and control healthy volunteers.

Regardless of whether rosacea patients or healthy control samples were examined, different microbiome composition results were obtained according to the sample type. In the principal component analysis diagram, the fecal, blood and skin microbiome results created three distinct groups (Figure 1A). On the heatmap diagram of bacterial genera, bacteria occurring in fecal, blood and skin samples were present with different abundances (Figure 1B). The genera *Cutibacterium, Corynebacterium* and *Neisseria* participated in high abundance among the members of the skin microbiome but were not or barely detectable in the stool and blood microbiome. The genus *Staphylococcus* was detected in some blood microbiomes, appeared in high abundance in the skin microbiomes and was absent in the stool microbiomes.

**Figure 2:**
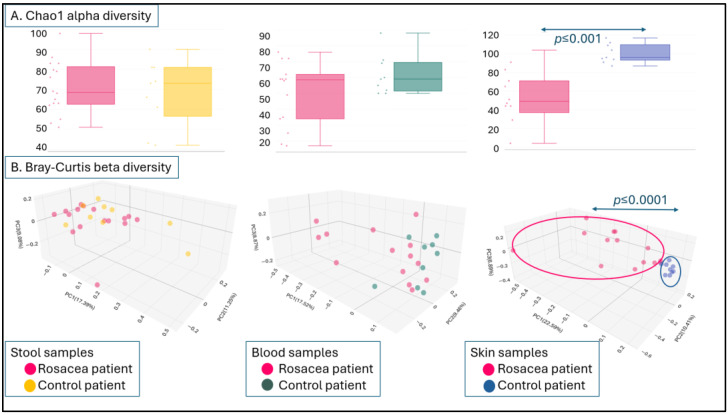
Comparison of Chao1 alpha diversity (**A**) or Bray–Curtis beta diversity (**B**) of stool, blood and skin samples from the rosacea patients and control patients.

Comparing the Chao1 alpha diversity results of rosacea patients and control patients, no significant difference was found among the stool or blood samples. No significant differences in beta diversity could be verified when comparing the samples of the rosacea patients and healthy controls, regardless of whether they were obtained from the stool or blood samples. In the Bray–Curtis principal coordinate beta diversity representation, the blood data of rosacea patients and control subjects appeared to be different, but the difference was not statistically significant (*p* = 0.074). The skin microbiome Chao1 alpha diversity of the rosacea patients was significantly lower than that of the healthy controls (*p* ≤ 0.001). As shown in the Bray–Curtis beta diversity principal coordinate analysis figure below, the samples of rosacea patients and the control samples are organized into two significantly different sets (*p* ≤ 0.0001) (Figure 2A,B).

**Figure 3:**
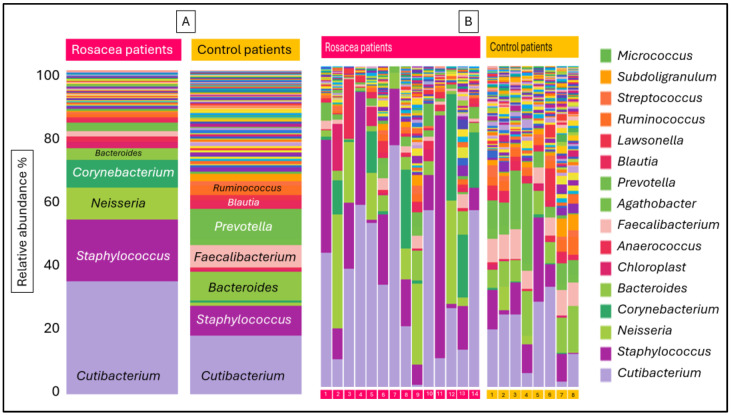
The genus relative abundance in the composition of the skin microbiome of rosacea patients and control individuals, aggregated by cohort (**A**) and individual data (**B**) by stacked bar representation.

On one hand, the difference between the skin microbiome of rosacea patients and healthy controls is made up of many more taxa, resulting in high alpha diversity. On the other, due to the more colorful composition of the healthy microbiome, the relative abundance of the most characteristic skin microbiome components appeared to be lower in the healthy microbiome compared to the rosacea microbiome. Additionally, there are taxa that are either only characteristic of healthy or only rosacea affected skin. The difference between the main microbiome components of the two cohorts (Figure 3A) is indicated by median abundance values of the cohorts, but due to the large deviation of the abundance values per individual (Figure 3B), a significant difference could only be verified for a few genera. Of the genus abundances characteristic of rosacea patients, *Neisseria* and *Corynebacterium* were significantly higher, while *Corynebacterium* and *Staphylococcus* were not significantly higher than in healthy patients. The abundances of genera *Bacteroides*, *Faecalibacterium*, *Prevotella*, *Blautia*, *Ruminococcus* and *Subdoligranulum* are all significantly higher in the skin microbiome of the healthy patients. The genera *Cutibacterium*, *Neisseria*, *Staphylococcus* and *Corynebacterium* were missing from the stool samples, and they were present with low abundance in only a few blood samples. The other six genera with significantly different abundances in the skin microbiome appeared with similar abundances in the negative control and rosacea stool samples, and no significant difference was observed. Comparing the control and rosacea blood samples, significant difference between the abundance of the genera *Ruminococcus* and *Subdoligranulum* was found (Table 2).

**Figure 4:**
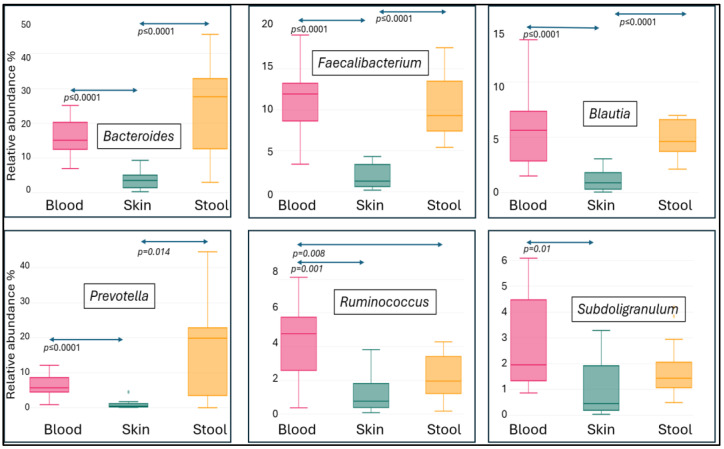
Abundance distribution of *Bacteroides*, *Faecalibacterium*, *Blautia*, *Prevotella*, *Ruminococcus* and *Subdoligranulum* genera among the different sample types.

There was no significant difference in the abundance distribution of blood and feces for the genera *Bacteroides*, *Faecalibacterium*, *Blautia* and *Prevotella*, but the abundance of all four genera in skin samples was significantly lower than in blood or feces. The abundance of *Ruminococcus* and *Subdoligranulum* in the blood samples was higher than in the stool samples and significantly higher than in the skin samples (Figure 4).

**Figure 5:**
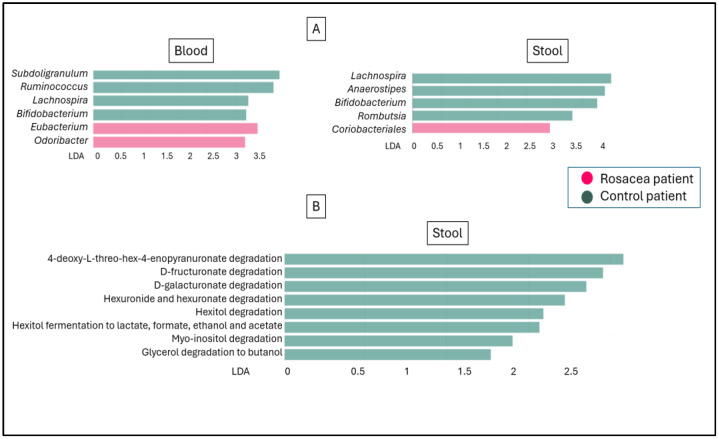
LEfSe bar chart; visual representation of discriminative features of genera abundances among the control and rosacea blood and stool samples (**A**), and discriminative biochemical pathways in stool samples (**B**). LDA is the linear discriminant analysis score.

Although there were no significant differences in abundance in the main components when comparing rosacea vs. control stool samples or rosacea vs. control blood samples, statistically significant differences in abundance could be demonstrated for some less common genera. The control patients’ blood contained significantly more *Subdoligranulum*, *Ruminococcus*, *Lachnospira* and *Bifidobacterium* and contained significantly less amounts of *Eubacterium* and *Odoribacter.* In control stool samples, *Lachnospira*, *Anaerostipes*, *Bifidobacterium* and *Rombutsia* occurred with significantly higher abundances, while *Coriobacteriales* had a significantly lower abundance. LEFSe analysis can be used to determine a characteristic that is present in a significant amount within a given group, and that is discriminative against the other group. Comparing the biochemical activities of healthy and rosacea samples, only the biochemical pathways of the control group stool samples met the expected LDA and significance levels in the LEFSe analysis. Predicting and profiling the functional capabilities and quantities of the microbial communities showed significantly more active polysaccharide degradation metabolic pathways in the gut microbiome of control patients. Metabolizing fructuronate, galacturonate and other active pathways in the gut microbiome of control patients contributes to the production of short-chain fatty acids (SCFAs) like acetate and butyrate. These SCFAs are known to have anti-inflammatory properties (Figure 5).

**Figure 6:**
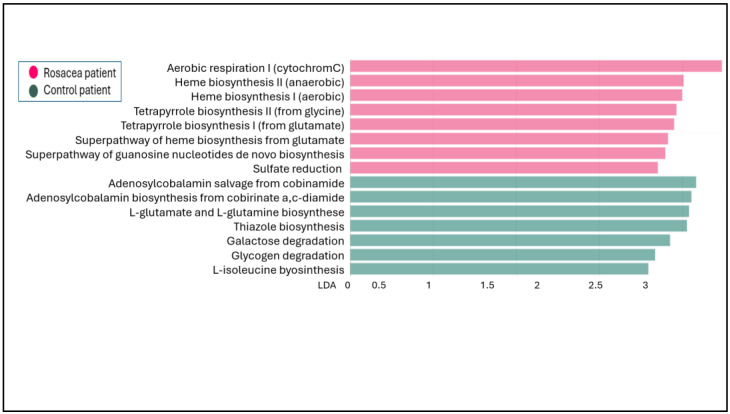
LefSe analysis of metabolic pathways of the rosacea and control skin microbiome. LDA is the linear discriminant analysis score.

Comparing the biochemical activity of the healthy and rosacea skin microbiomes, 46 noteworthy biochemical pathways were significantly more common in rosacea patients, and 72 representative pathways were present in healthy subjects. In Figure 6, only the different characteristic biochemical pathways are presented for which the significance level is between *p* ≤ 0.001 and *p* = 0.01. The skin microbiome of rosacea patients is distinctly more active in several biochemical pathways involved in heme biosynthesis. Heme is an iron-containing tetrapyrrole, the biosynthesis of which is also part of the heme synthesis pathway. Heme acts as a potent pro-inflammatory molecule. Another prominent biochemical activity of the rosacea microbiome is sulfate reduction. The excessive hydrogen sulfide (H_2_S) production by sulfate-reducing bacteria plays a role in inflammation. In contrast, the healthy skin microbiome shows remarkable activity in adenosylcobalamine biosynthesis. Adenosylcobalamin is a B12 derivative, similarly to hydroxycobalamin, that has been shown to effectively reduce facial flushing and erythema. Increased thiazole production by the healthy skin microbiome may also have a protective function, as thiazole derivatives are known to have anti-inflammatory efficacy. Increased L-isoleucine production in the control group’s microbiome may also have a protective role. L-isoleucine exhibits anti-inflammatory effects by modulating the activity of pro-inflammatory cytokines and pathways.

## 4. Discussion

With the development of diagnostic methods, a great overview is regularly presented regarding the pathogenesis of rosacea and the various microbes involved. As early as 1932, it was shown using light microscopy that *Demodex* mites are present in increased numbers on the skin of rosacea patients and play a role in the development of the symptoms [23]. Using aerobic and anaerobic culture methods, additional bacteria have been identified that may be involved in the development of rosacea, including *Staphylococcus epidermidis*, *Bacillus oleronius* or *Cutibacterium acnes* [24]. The use of next-generation sequencing technology has opened new perspectives for detecting changes in the composition of bacterial communities. Analysis of 16S ribosomal RNA, superior to culture-based studies, allowed for the investigation of the composition of the skin, stool and blood microbiome. Several studies have documented differences in the skin and fecal microbiome between healthy individuals and patients with rosacea but have reported varying and sometimes contradictory results. Yun et al. found that rosacea is associated with an aberrant blood microbiome, comparing the blood microbiomes of women with and without rosacea [17]. Our current study looks simultaneously for differences in the skin, blood, and stool samples from healthy and rosacea individuals and looks for correlations in the composition of the skin, blood and stool microbiome within an individual.

The skin microbiome alpha and beta diversities we examined were also significantly different between rosacea and non-rosacea patients. Similar to Thompson’s results [14], we demonstrated significantly lower skin microbial alpha diversity in rosacea subjects. On the contrary, Wang and coworkers found increased alpha diversity in the skin microbiome of rosacea patients [25], while other reports do not demonstrate a significant difference in alpha diversity [12,13]. Only our study shows significant difference in the beta diversity between the skin microbiome of rosacea patients and controls. In the samples of our rosacea patients, we found an increased abundance of *Staphylococcus* and *Cutibacterium* as well as significantly increased abundance of the *Neisseria* and *Corynebacterium* genera. Several studies have confirmed an increase in *Corynebacterium* [13] and *Staphylococcus* [25,26,27] abundances, but there are also reports that have found either supportive [14,27] or contradictory [13,25] results in the change in *Cutibacterium* abundance. The high abundance of the *Neisseria* genus was also confirmed earlier [27]. Within the *Staphylococcus* genus, *S. epidermidis* is a beneficial member of the normal flora of the skin, so the pathogenicity of its higher abundance in rosacea skin is questionable. Since the temperature of facial skin in rosacea patients is warmer than that of healthy individuals, and *S. epidermidis* strains grown at higher temperatures secrete different proteins in different amounts, the pathogenic role of these proteins is likely to be justified [28]. The different results of *Cutibacterium* abundance can be interpreted in light of the fact that among the different *C. acnes* strains there are some that have protective and some that have pathogenic roles. However, as a beneficial effect, by occupying niches on the skin, *Cutibacterium* prevents the colonization of pathogenic microorganisms. Additionally, propionic acid and other metabolic products help maintain skin pH and inhibit the growth of harmful bacteria. Certain strains of *C. acnes* possess virulence factors that enhance their ability to cause disease; biofilm formation capabilities and the production of inflammatory mediators can trigger host immune responses, leading to tissue damage [29]. The highly active metabolic pathway due to tetrapyrrole and heme synthesis of the skin microbiome of our rosacea patients results in the development of high heme concentrations. High heme production promotes inflammation via toll-like receptors (TLRs). The activation of TLRs lead to the production of pro-inflammatory cytokines and reactive oxygen species (ROS) in immune cells such as macrophages and endothelial cells [30]. This activation can promote inflammation in rosacea patients. Increased H_2_S production can be predicted due to the predominance of sulfate-reducing bacteria in the skin microbiome of our rosacea patients. H_2_S plays a complex role in inflammation, acting as both pro-inflammatory and anti-inflammatory mediator, depending on the context. Chen et al. found increased H_2_S production in the gut microbiome of their rosacea patients, a process that compromises epithelial barrier functions through the impairment of colonic butyrate oxidation and subsequent disruption of epithelial permeability [16,31]. In contrast, healthy skin microbiome shows outstanding activity in the biosynthesis of adenosylcobalamine, a B12 derivative, like hydroxycobalamin, which has been shown to effectively reduce facial flushing and erythema in rosacea patients [32]. Our research only predicts increased adenosylcobalamin production in the non-rosacea skin microbiome, while other researchers detected increased cobalamin transport associated with the fecal microbiome of rosacea patients [16]. Since it is not well established whether the vitamin B complex supplements triggered or worsened rosacea symptoms [33], further research is needed to understand the relationship between vitamin B derivatives and the pathogenesis of rosacea. Increased thiazole and L-isoleucine production by the non-rosacea healthy skin microbiome may also have a protective function, as thiazole derivatives and L-isoleucine are known to have anti-inflammatory efficacy by modulating the activity of pro-inflammatory cytokines and pathways [34,35]. The skin microbiome of the control subjects we examined, in contrast to the rosacea patients, can produce anti-inflammatory metabolites such as adenosylcobalamin, thiazole or L-isoleucine. The excessive anti-inflammatory metabolite-producing bacteria (*Faecalibacterium*, *Prevotella,* etc.) detected in healthy subjects are anaerobic bacteria not used for skin surface treatment. However, numerous studies have confirmed the beneficial effects of bacteria that can also be used as probiotics on the skin surface, such as *Lactobacillus rhamnosus*, *L. paracasei*, *L. acidophilus* and *L. plantarum*, due to their anti-inflammatory effect [36,37].

No significant difference in either alpha or beta diversity was demonstrated in the fecal microbiome of our rosacea and healthy patients in our study. The results of the alpha diversity tests are also contradictory; Nam et al. found no significant difference [15]; Chen’s research group showed a significantly lower alpha diversity [16], and Moreno-Arrones et al. confirmed a significantly higher alpha diversity in the case group compared to the control group [38]. The results of both studies are consistent and point to a statistically significant inter-sample beta diversity of the gut microbiome. The result of alpha diversity is a measure of the richness and evenness of microbial taxa within a community, and beta diversity compares the microbiological composition of various communities. The results may differ because each study included a relatively small number of rosacea patients; the number of patients studied varied between 11 and 15 per research group. Genera present in significant amounts in the stool but differing across cohorts were *Coriobacteriales* with higher abundance in rosacea patients and *Lachnospira*, *Anaerostipes*, *Bifidobacterium* and *Rombutsia* in the control group. Other reports claim that the abundance of other bacterial taxa is significant; different microbiome compositions show similar biochemical activities when comparing rosacea and control samples. In agreement with an earlier report [16], we also demonstrated increased carbohydrate breakdown due to fructuronate and galacturonate metabolisms in the fecal microbiome of the control group and its deficiency in rosacea patients. Based on the predictive metabolic activity differences, it can be assumed that significantly less anti-inflammatory SCFAs are produced in rosacea patients compared to the gut microbiome of the non-rosacea control group.

We could not identify significant differences in the blood microbiome alpha and beta diversity between rosacea and non-rosacea subjects. While there was no significant difference in alpha diversity in the study by Yun et al. [17], their study included 10 rosacea and 30 rosacea-free subjects, they found significant differences in beta diversity. The above-mentioned study, which investigated blood microbiome composition in rosacea subjects, found *Rheinheimera* as the genus with the most prominently different higher abundance. This study examined the blood of Korean individuals; neither our current study nor previous studies detected *Rheinheimera* DNA in the blood of any Hungarian individuals [39,40]. Microbial products responsible for increased inflammation in the gut may also reach the skin via the blood, but similarly, the blood might constitute a link between bacteria that make up the gut and skin microbiome. To the best of our knowledge, this is the first study to simultaneously detect differences in the fecal, blood and skin microbiome in individuals with and without rosacea. The bacterial genera with different abundances, which caused significant difference in the skin microbiome of the two study groups, did not show any differences when comparing blood and stool samples. Rosacea is a disease with a complex pathomechanism and is also influenced by previously described changes in the gut and skin microbiome composition. Having said that, the role of blood bacterial translocation in this process could not be confirmed by our current study.

Our study had several limitations, including the small sample size and the consequent inability to group our data according to rosacea subtypes.

## 5. Conclusions

The skin microbiome characteristics of rosacea patients differ from those of healthy individuals, either in alpha or beta diversity, or in the higher abundances of bacterial genera *Staphylococcus*, *Corynebacterium*, *Cutibacterium* and *Neisseria*. We could not detect similar significant genus abundance differences in the fecal and blood microbiomes, thus at this point, the transfer of gut microbiome changes via the blood to the skin cannot be confirmed. Importantly, the products of metabolic pathways predicted from the composition of the skin and gut microbiome of rosacea patients have pro-inflammatory effects, while the products of biochemical activity of the skin and gut microbiome of rosacea-free individuals have anti-inflammatory effects. These findings suggest microbiome-targeted treatments could improve rosacea management.

## Figures and Tables

**Table 1:** Characteristics of the study participants’ number, gender and median age in year and interquartile range (IQR), type of rosacea.

	Number of Participants, Gender (m/f)	Age, Year (Median + IQR)	Type of Rosacea
Rosacea patients	18, (4/14)	42, IQR: 15	14 PPR, 1 ETR, 3 PPR + ETR
Control patients	9, (2/7)	39, IQR: 13.5	NA

**Table 2:** Comparison of the median relative abundance of taxa at the genus level in the skin samples of rosacea patients and control subjects. Significantly higher abundance and *p* values are highlighted in bold.

Sample Type	Genus	Rosacea Patients	Control Patients	*p* Value
Skin	*Cutibacterium*	35.14	20.93	0.088
	***Neisseria***	**18.65**	1.35	**0.023**
	*Staphylococcus*	13.97	8.82	0.152
	***Corynebacterium***	**10.56**	0.21	**0.015**
	***Bacteroides***	2.94	**7.49**	**0.001**
	***Faecalibacterium***	1.28	**7.42**	**<0.001**
	***Prevotella***	0.47	**6.75**	**0.001**
	***Blautia***	0.83	**2.81**	**0.001**
	***Ruminococcus***	0.69	**2.48**	**0.001**
	***Subdoligranulum***	0.28	**1.71**	**0.017**
Blood	***Ruminococcus***	4.7	**7.13**	**0.032**
	***Subdoligranulum***	1.95	**4.65**	**0.033**

## Data Availability

The datasets generated and analyzed during the current study are available in the SRA repository: SRA/PRJNA 1189573/www.ncbi.nlm.nih.gov (accessed on 22 November 2024).
